# Morphologic and Molecular Characterization of Adrenals and Adrenal Rest Affected by Congenital Adrenal Hyperplasia

**DOI:** 10.3389/fendo.2021.730947

**Published:** 2021-09-20

**Authors:** Vipula Kolli, Isabela Werneck da Cunha, SunA Kim, James R. Iben, Ashwini Mallappa, Tianwei Li, Alison Gaynor, Steven L. Coon, Martha M. Quezado, Deborah P. Merke

**Affiliations:** ^1^National Institutes of Health Clinical Center, Bethesda, MD, United States; ^2^Laboratory of Pathology, National Cancer Institute, Bethesda, MD, United States; ^3^The Eunice Kennedy Shriver National Institute of Child Health and Human Development, Bethesda, MD, United States

**Keywords:** adrenal insufficiency, congenital adrenal hyperplasia, testicular adrenal rest tissue, para-ovarian adrenal rest tissue, principal component analysis

## Abstract

**Introduction:**

Adrenocortical hyperplasia and adrenal rest tumor (ART) formation are common in congenital adrenal hyperplasia (CAH). Although driven by excessive corticotropin, much is unknown regarding the morphology and transformation of these tissues. Our study objective was to characterize CAH-affected adrenals and ART and compare with control adrenal and gonadal tissues.

**Patients/Methods:**

CAH adrenals, ART and control tissues were analyzed by histology, immunohistochemistry, and transcriptome sequencing. We investigated protein expression of the ACTH receptor (MC2R), steroidogenic (CYP11B2, CYP11B1, CYB5A) and immune (CD20, CD3, CD68) biomarkers, and delta-like 1 homolog (DLK1), a membrane bound protein broadly expressed in fetal and many endocrine cells. RNA was isolated and gene expression was analyzed by RNA sequencing (RNA-seq) followed by principle component, and unsupervised clustering analyses.

**Results:**

Based on immunohistochemistry, CAH adrenals and ART demonstrated increased zona reticularis (ZR)-like CYB5A expression, compared to CYP11B1, and CYP11B2, markers of zona fasciculata and zona glomerulosa respectively. CYP11B2 was mostly absent in CAH adrenals and absent in ART. DLK1 was present in CAH adrenal, ART, and also control adrenal and testis, but was absent in control ovary. Increased expression of adrenocortical marker MC2R, was observed in CAH adrenals compared to control adrenal. Unlike control tissues, significant nodular lymphocytic infiltration was observed in CAH adrenals and ART, with CD20 (B-cell), CD3 (T-cell) and CD68 (macrophage/monocyte) markers of inflammation. RNA-seq data revealed co-expression of adrenal *MC2R*, and testis-specific *INSL3, HSD17B3* in testicular ART indicating the presence of both gonadal and adrenal features, and high expression of DLK1 in ART, CAH adrenals and control adrenal. Principal component analysis indicated that the ART transcriptome was more similar to CAH adrenals and least similar to control testis tissue.

**Conclusions:**

CAH-affected adrenal glands and ART have similar expression profiles and morphology, demonstrating increased CYB5A with ZR characteristics and lymphocytic infiltration, suggesting a common origin that is similarly affected by the abnormal hormonal milieu. Immune system modulators may play a role in tumor formation of CAH.

## Introduction

Congenital adrenal hyperplasia (CAH) is a group of autosomal recessive disorders of the adrenal gland affecting cortisol biosynthesis, mostly due to 21-hydroxylase deficiency (21-OHD). Impairment of cortisol production results in lack of negative feedback to pituitary adrenocorticotrophic hormone (ACTH) production and consequent ACTH excess leads to adrenocortical hyperplasia. CAH manifests with a wide range of clinical and biochemical severities. Due to therapeutic advances in hormone replacement, patients live well into adulthood, although multiple comorbidities and adverse outcomes occur (1). One common disease-related manifestation, is tumor formation, with the development of adrenal tumors and adrenal rest tumors (ARTs).

ARTs are extra-adrenal-like masses with morphological and functional similarities to adrenocortical tissue (2, 3). ARTs are reported to be found in different organs, such as the testes (testicular adrenal rest tumor -TART), ovaries or para-ovaries (ovarian and para-ovarian adrenal rest tumor–OART and para-OART), liver (4), or spinal canal (5, 6). In 1883, ectopic adrenocortical tissue was described as accessory nodules in the mesovarium, mesosalpinx, broad ligament, and the wall of the fallopian tube and within the ovaries (7). Since then, less than 20 cases of OART and para-OART in CAH have been reported, with a range of symptoms, some causing ovarian dysfunction (8–11). Reports of OART and para-OART are rare, likely because they are not easily detected by regular imaging studies (12).

TARTs are commonly observed in men with CAH and, unlike OART, are easily detected by ultrasound (13–15). The prevalence of TART with CAH ranges from 14 - 89% based on the age of the cohort and the detection techniques used (16), and the prevalence increases during adolescence. Though these are not malignant, based on their location in the rete testis, TART can cause irreversible damage to the surrounding testicular tissue and may result in gonadal dysfunction and infertility (13). TART has been shown to be correlated with elevated ACTH levels in adult CAH males (17). TARTs are reported to be the most common cause of male infertility in CAH (3). Previous studies have described TARTs to be well-demarcated from surrounding testicular tissue and microscopically to contain large polygonal cells with abundant granular eosinophilic cytoplasm (16, 18, 19).

The etiology of TART and factors contributing to its origin and progression are not completely clear. Some studies support the concept that these benign tumors arise from pluripotent progenitor cells or from cells which are adrenal in origin which descend with the testis during embryogenesis (16, 20–22) and proliferate with ACTH stimulation (23). Clinical reports support this theory in that TART is found in high ACTH states and a decrease in TART size is observed with high dose glucocorticoid treatment and suppression of ACTH (24, 25). It has been suggested that TART is mainly observed in poorly controlled CAH patients with elevated ACTH contributing to the development and pathogenesis of TART, however its appearance is also noted in well-controlled patients and TART is not found in all CAH males with poor hormonal control (26–28). TART is also observed in acquired adulthood conditions such as Cushing’s disease, Nelson’s syndrome and Addison’s disease, suggesting that the duration and degree of ACTH exposure might play a role in the proliferation and transformation of cells into TART, along with other unknown contributing factors (25, 29, 30).

Adrenal tumor formation and adrenal hyperplasia are common long-term complications of CAH. Increased adrenal volume and adrenocortical hyperplasia are associated with the development of comorbidities such as hypogonadism, and metabolic risk factors in patients with CAH (31). In general, in CAH, adrenal hyperplasia is associated with higher adrenal steroid levels and the development of adrenal tumors. To our knowledge, detailed morphologic and molecular characterization of CAH-affected adrenals has not been performed.

Understanding the pathogenesis and functional features of tumor formation is essential in developing treatment strategies. This is the first study describing the structural morphology of the cells residing in adrenals from patients with CAH in comparison with ART. In addition, we report gene expression studies. This study provides a comprehensive characterization of CAH-affected adrenals and ART in relation to control tissues, thus providing insight into disease-specific tissue transformation.

## Patients and Methods

### Patients/Clinical Aspects

Patients with CAH were enrolled in the Natural History study at the National Institutes of Health Clinical Center (NCT#00250159) and studies were approved by the National Institute of Health (NIH) Institutional Review Board. The majority of patients (five out of six) were receiving glucocorticoid treatment at the time of surgery, and all patients had a history of years of noncompliance or undertreatment. All adult patients and parents of participating minors provided informed consent and all minors at least 7 years old provided written assent. Clinical and radiological characteristics of the six subjects are summarized in **Table 1** (12, 32–34). One of each, control adrenal, testicular, and ovarian tissues were obtained from a de-identified tissue bank at the National Institutes of Health, Pathology Department, Bethesda, MD. All tissues are reviewed by a pathologist and deemed appropriate to use as control tissues. The adrenal tissue was from a 30 year-old woman who died from an acute pulmonary hemorrhage. The age and cause of death of the gonadal tissue donors are unknown.

**Table 1:** Clinical, genetic and radiological findings of six patients with adrenal and/or adrenal rest masses.

Patient No.		CAH-1	CAH-2	CAH-3	CAH-4	CAH-5	CAH-6
Sex		F	F	F	F	M	F
Age at CAH presentation		Birth	8 days	2 years	6 weeks	3.6 years	Birth
CAH type		21-OHD (32)	21-OHD (12)	21-OHD (33)	21-OHD	P450scc Deficiency (34)	21-OHD
Genotype		intron 2 IVS2-13A/C>G/p.R483P	homozygous exon 6 cluster: p.I236N, p.V237E, p.M239K	p.I172N/30 kb deletion	30kb deletion/30kb deletion	I279Yfs*10/p.E314K	intron 2 IVS2-13A/C>G/30 kb deletion
Phenotype		SV	SW	SV	SW	NC	SV
Sample tissue		Adrenal	Adrenal, Para-OART	Adrenal	Adrenal	TART	Adrenal
Radiology findings prior to surgery		CT adrenals: lobulated, enhanced L adrenal mass measuring 10 x 3 x 7 cm compressing the L kidney	CT adrenals (at age 16): markedly enlarged bilateral adrenals	CT adrenals: bilateral moderately hyperplastic adrenals with a L adrenal gland nodule	CT adrenals: Three masses from L adrenal, largest 6.2 x 1.5 cm and R adrenal mass arising from the medial limb measuring 1.9 x 1.8 cm - bilateral adrenal masses suggestive of myelolipoma	Scrotal U/s (at age 16): R testis measured 4.5 x 2 cm with three hypoechoic lesions measuring 2.1 x 1.0 cm, 1.2 x 1.0 cm, 0.4 x 0.3 cm.	CT adrenals: Fat containing complex L adrenal mass measuring 13 x 12 x 11 cm suggestive of myelolipoma. L kidney displaced inferiorly
			^18^F-PET/CT with Cosyntropin 250 µg injection			L testis measured 4.3 x 2.7 cm - two hypoechoic lesions: 2.0 x 1.0 cm, 1.3 x 1.0 cm with diffuse peripheral calcification	
			(at age 32): three areas of active uptake near both ovaries				
Indication for surgery		Abdominal pain secondary to large L sided adrenal mass	Virilization with primary amenorrhea at age 16, had marked adrenal enlargement and underwent bilateral adrenalectomy at age 17. At age 32 presented with hyperandrogenism. Workup consistent with ectopic adrenal rest tumors near the ovaries	Poor disease control with secondary amenorrhea and patient desired fertility	Incidentally identified adrenal masses for work up chronic lower abdominal pain	Incidentally identified testicular mass of unknown etiology (prior to CAH diagnosis)	Abdominal pain secondary to large L sided adrenal mass R adrenal – bilateral flank pain and hematuria
Age at surgery (years)		29	32	21	58	17	42
Surgical procedure		L-sided adrenalectomy	Bilateral adrenalectomy, excision of paraovarian adrenal rest tumors	Bilateral adrenalectomy	L-sided adrenalectomy	L testicular exploration with tumor enucleation	Bilateral adrenalectomy
Medications At Surgery	Glucocorticoid^¥^ equivalent- dose (mg/day)	None- was off meds for 13 years	30^#^	40^$^	15 ^Ω^	12.5	40*
	Fludrocortisone (µg/day)		100	150	None	100	150

F, female; M, male; CAH, congenital adrenal hyperplasia; 21-OHD, 21-hydroxylase deficiency; P450scc, cytochrome P450 side chain cleavage; kb, kilobase; SV, simple-virilizing; SW, salt-wasting; NC, non-classic; CT, computed tomography; L, left; ^18^F-FDG PET, ^18^F-fluorodeoxyglucose positron emission tomography; U/s, ultrasound; R, right.

^¥^Glucocorticoid-equivalent dose, hydrocortisone x 1, and dexamethasone x 80;

^#^dexamethasone 0.375 mg once daily;

^$^dexamethasone 0.25 mg twice daily;

*dexamethasone 0.5 mg once daily;

^Ω^hydrocortisone twice daily.

### Sample Preparation

Formalin–fixed paraffin–embedded (FFPE) tumor sample blocks of one testicular adrenal rest, one para-ovarian adrenal rest, and five adrenal glands from patients with CAH were evaluated, along with control adrenal, testicular, and ovarian tissues.

### Histopathologic Analysis

Multiple tissue sections of 5 µm thickness were made from fixed paraffin embedded tissue (CAH adrenal, adrenal rest, control adrenal, control ovary, and control testis) and were mounted on Superfrost Plus slides (Erie Scientific). Hematoxylin and Eosin (H&E) staining was performed using a Leica CV5030 autostainer (Leica). Immunohistochemistry (IHC) was performed using antibodies specific to delta-like non-canonical notch ligand 1 (DLK1) (ab21683 (1:2500), abcam, MA, USA) and, adrenocorticotropic hormone receptor or ACTH receptor (MC2R) (LS-C164069 (1:500), LifeSpan Biosciences, WA, USA). To evaluate the type of cells present, tissue sections were stained with zone-characteristic immunohistochemical staining: CYP11B2 (zona glomerulosa: ZG), CYP11B1 (zona fasciculata: ZF) and CYB5A (zona reticularis: ZR) using the following antibodies: Cytochrome P450 11-beta hydroxylase: member 1 (CYP11B1) (MABS502 (1:4000), MilliporeSigma, MA, USA), member 2 (CYP11B2) (MABS1251 (1:1000), MilliporeSigma, MA, USA), Cytochrome b5, type A (CYB5A) (Acris AM31963PU-N (1:500), MilliporeSigma, MA, USA). Staining with a panel of prediluted antibodies (Roche Tissue Diagnostics, MA, USA), specific to inflammation markers was performed using CD3 (790-4341: clone 2GV6), CD20 (760-2531: clone L26), and CD68 (790-2931: clone KP-1). CD20 and CD3 are specific for B and T-lymphocyte surface proteins respectively, and CD68 is specific for macrophage/monocyte histiocytes. While performing the IHC stainings, tonsil tissue was used as the positive control for CD20, CD3 and CD68, adrenal tissue was used for MC2R, CYP11B1, CYP11B2, CYB5A, whereas placenta was used for DLK1 stainings. Similar procedures were performed on the slides without primary antibody, serving as the reagent negative controls. H&E and IHC slides were viewed at X40, X100, X200 and X400 magnification. All tissue sections were first observed under a light microscope (Nikon) and subsequently examined on full HD camera (ToupCam XCAM, Nikon) before the stainings were performed.

### RNA Isolation and Sequencing

For each sample, eight 5µm cut unstained FFPE sections were used to isolate total RNA (CAH-1, CAH-2, and CAH-3 from **Table 1**) and ART (CAH-2/para-OART and CAH-5/TART). Entire sections were used without microdissecting zones. Total RNA was extracted using the Qiagen RNeasy FFPE kit (QIAGEN, Valencia, CA) according to the manufacturer’s instructions. RNA yield, quality, and size distribution were determined using the RNA 6000 Nano Assays using a 2100 Agilent Bioanalyzer (Agilent Technologies, Santa Clara, CA). Sequencing libraries were constructed using SMARTer Stranded Total RNA-Seq Kit v2-Pico Input Mammalian (Takara Bio, Mountain View, CA) modified by incorporating an initial DNAse treatment step using heat-labile HL-dsDNase according to the manufacturer’s protocol (ArcticZyme, Norway). Libraries were indexed and sequenced on a HiSeq-2500 (Ilumina, San Diego, CA). RNA-seq data were aligned to the reference human GRCh38 genome assembly using RNA-STAR 2.7.1a against GENCODE human v32 gene index definitions. Quantification of the aligned reads was performed using Subread feature Counts 1.6.4 using Gencode v32 gene definitions. The output files were used to identify and compare for differential expression tests of genes using DESeq2.

### Clustering Analysis

Unsupervised hierarchical clustering and principle component analysis (PCA) were performed on the complete set of normalized (transformed) data to understand the correlation between the samples.

## Results

### Histological Features of Adrenal Rest Tissue

**Figure 1:**
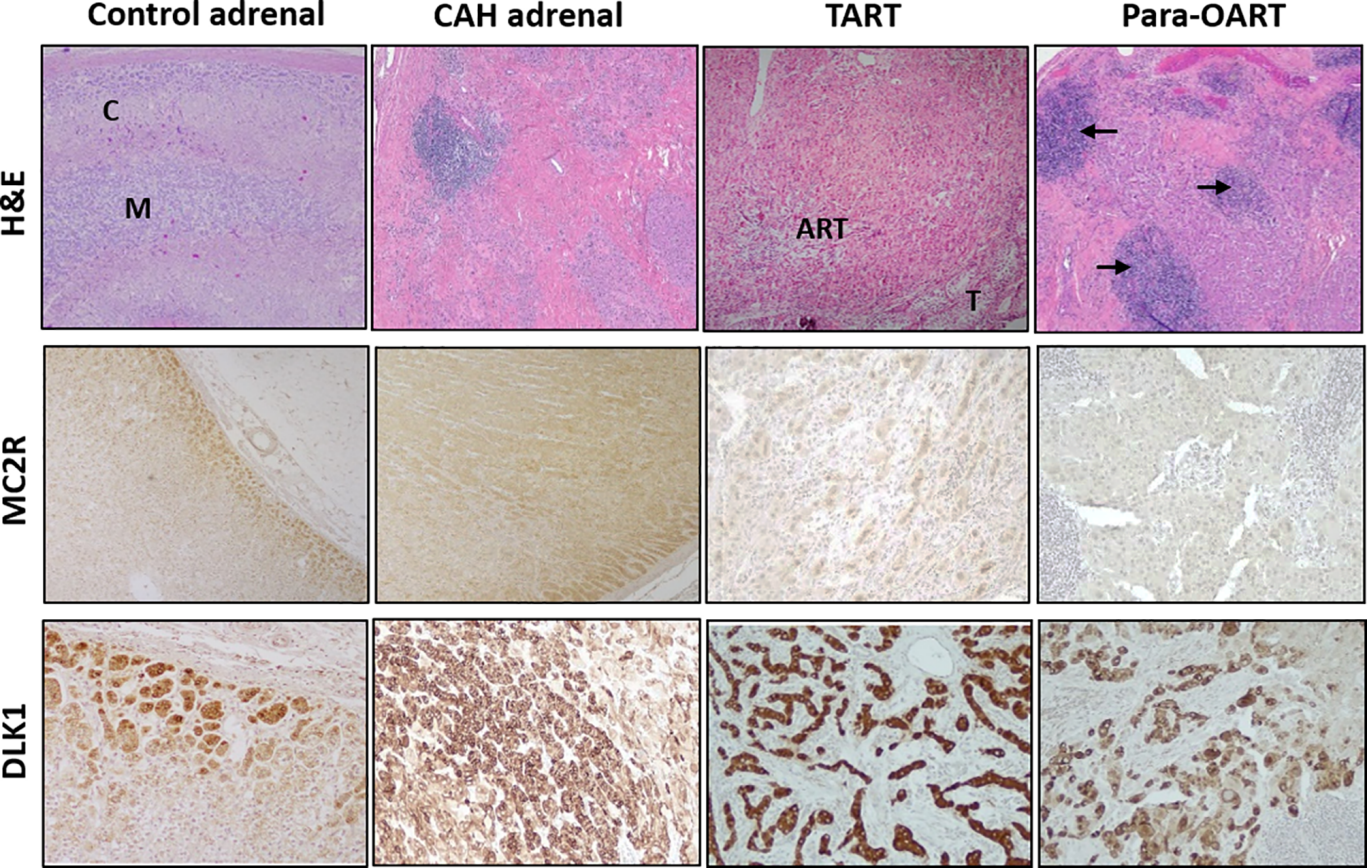
Histological comparison of adrenal rest tumor tissue (ART) and adrenal glands from CAH patients. Hematoxylin and eosin (H&E) staining with low power (40X) observation of control adrenal, CAH adrenal and ARTs shows: control adrenal glands with clear zonation including distinct cortical (C) and medullary areas (M), CAH adrenal with hyperplastic adrenocortical cells and no clear zonation, testicular adrenal rest tissue (TART) consisting of eosinophilic epithelioid cells with abundant cytoplasm and testicular seminiferous tubules (T) in the periphery and para-ovarian adrenal rest tissue (Para-OART) cells with large nuclei and with lymphoid aggregates in between (arrows). Immunohistochemistry images show positive staining for adrenal- MC2R (adrenocorticotrophic hormone receptor: ACTHR) and DLK1 (delta like non-canonical notch ligand 1) protein expression in control adrenal, CAH adrenal (representative sample) and also in adrenal rest tissue (Original magnification X40 for MC2R and X100 for DLK1).

The CAH adrenal showed a disorganized hyperplastic thickened adrenal cortical region with lack of the normal adrenal zonation (**Figure 1**) and florid lymphoid content in some sections. Microscopically, the TART appeared as fragmented numerous nodular aggregates of ectopic adrenal-like tissue mostly in a disorganized pattern (**Figure 1**). Para-OART consisted of abnormal cells with very large nuclei with oncocytic pink stained cytoplasm and no malignant characteristics (**Figure 1**). The structural histology of the ART and the adrenal tissue from CAH patients were similar. IHC staining showed adrenal-specific MC2R protein present in CAH adrenals, TART, and para-OART, similar to control adrenals (**Figure 1**). DLK1 staining was positive in control adrenal, CAH adrenal, and ART (**Figure 1**), and was weakly positive in control testis, but negative in control ovary (**Supplementary Figure 1**). Very strong DLK1 staining and mild to strong MC2R staining was observed in all of the ART and adrenal samples.

### Morphological Evaluation and Steroidogenic Immunohistochemical Analysis

**Figure 2:**
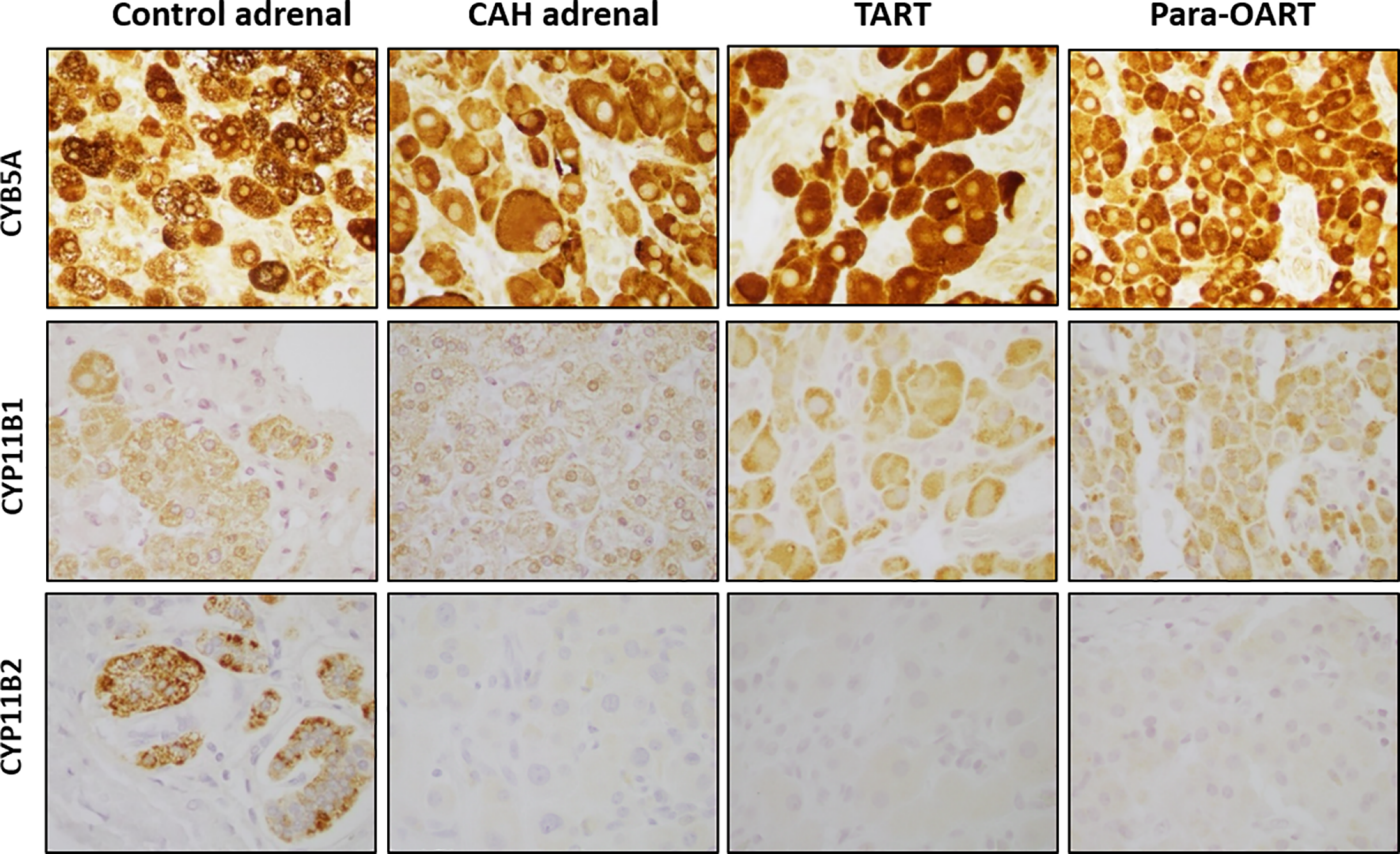
Steroidogenic immunolabeling in control and CAH adrenals,TART and para-OART. Immunohistochemical staining of control adrenal shows positive staining for all three zone-characteristic proteins. ART and representative CAH adrenals display strong staining of zona reticularis-characteristic CYB5A, a marker of androgen production, positive staining but less presence of CYP11B1, and negative staining for CYP11B2 proteins. Two of five CAH adrenals had minimal CYP11B2 staining (not shown). Interstitial/vascular spaces with fibroblasts, vessels and other structures are negatively stained. (Original magnification X400).

Positive staining for CYB5A, CYP11B1 and CYP11B2 corresponding to the three adrenal cortical zones, ZR, ZF and ZG, respectively, was found in the control adrenal tissue (**Figure 2**). Positive CYB5A staining was also present in both control testis and control ovary, reflecting its role in androgen production (**Supplementary Figure 1**). Three out of five CAH adrenal samples showed high expression of CYB5A indicated by a very strong positive (~95%) staining, with less than 10% of cells with CYP11B1 positive staining, and lack of CYP11B2 staining, indicating the absence of normal ZG cells. The remaining two CAH adrenals also showed strong positive staining for CYB5A, and minimal CYP11B1 staining, but with some cells positive for CYP11B2 expression. Immunohistochemical markers of adrenal rest (both TART and para-OART) revealed very strong positive staining (90-95%) for CYB5A, less prevalent positive staining (5-10%) for CYP11B1 and negative staining for CYP11B2 (**Figure 2**).

### Immune-Specific Response

**Figure 3:**
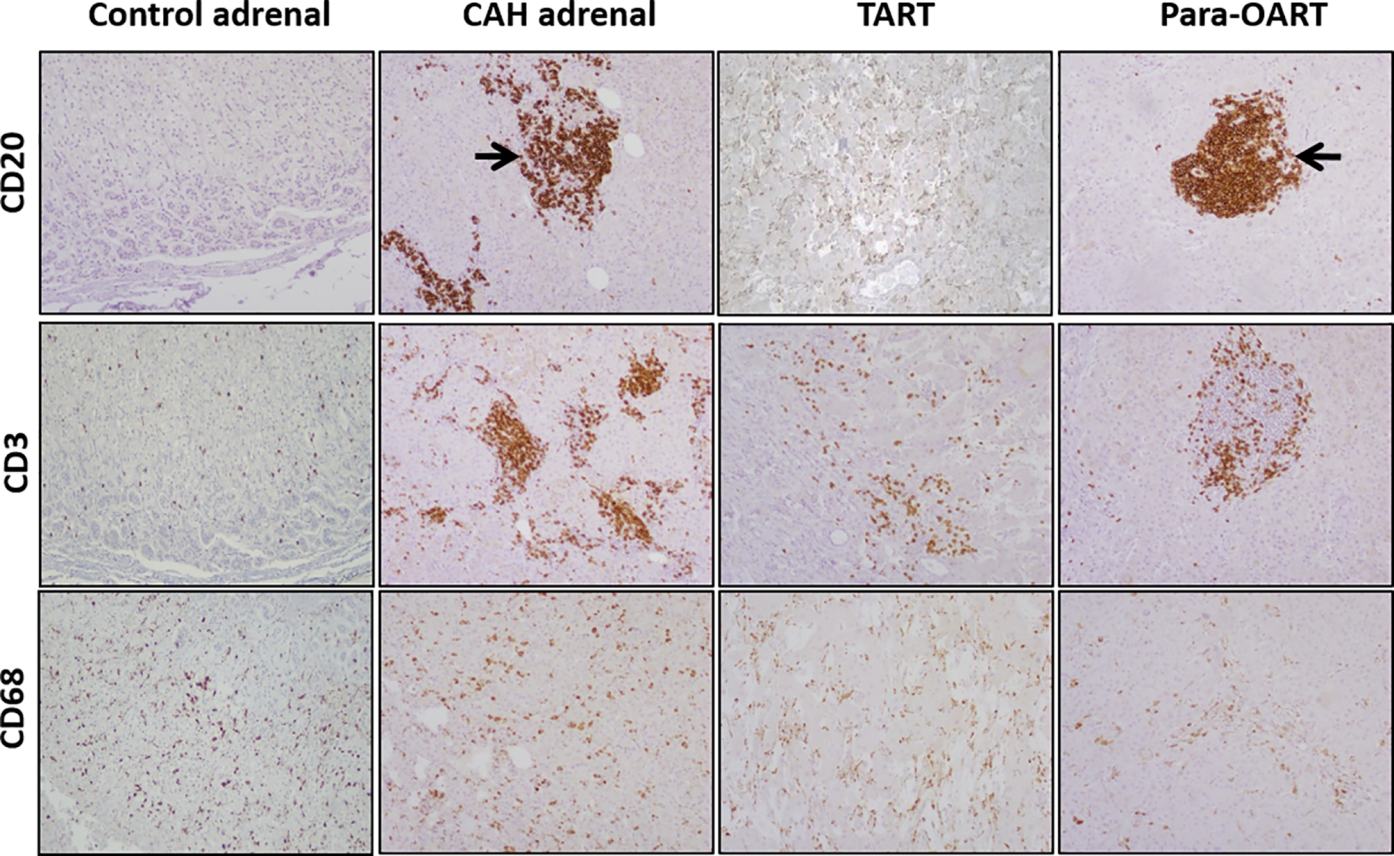
Immunohistochemical staining for mono nuclear cell infiltration, labeling with CD20, CD3, CD68 in control and, CAH adrenals and TART and Para-OART. Immunohistochemical staining of ART and CAH adrenals with lymphocytic markers CD20 (B-lymphocytes), CD3 (T-lymphocytes) and CD68 (histiocytes) displays an elevated expression in lymphocytic aggregates in the adrenal parenchyma in CAH adrenals and nodular lymphocytic aggregates in para-OART (arrows). CD68 shows scattered histiocytes through the cortex or within the cortical cells (Original magnification X100).

CAH adrenals and para-OART contained islets of cells comprised of mature and precursor myeloid lineage. Fibrotic tissue with adrenocortical-like cells and aggregated nodules were observed (**Figures 1**, **3**). B-lymphocytes were localized at the nodules in these tissues. Very dense B- and T-cell lymphocytic infiltration was observed in three out of five CAH adrenals and in the para-OART with positive staining of CD20 and CD3 inflammatory markers. Positive staining for CD68 specific for (macrophages/monocytes) histiocytes was observed in lymphoid aggregates and scattered through the cortex (**Figure 3**). CD20, CD3 and CD68 showed a mildly strong but consistent expression in TART (**Figure 3**).

### RNA-Sequencing Analysis

**Figure 4:**
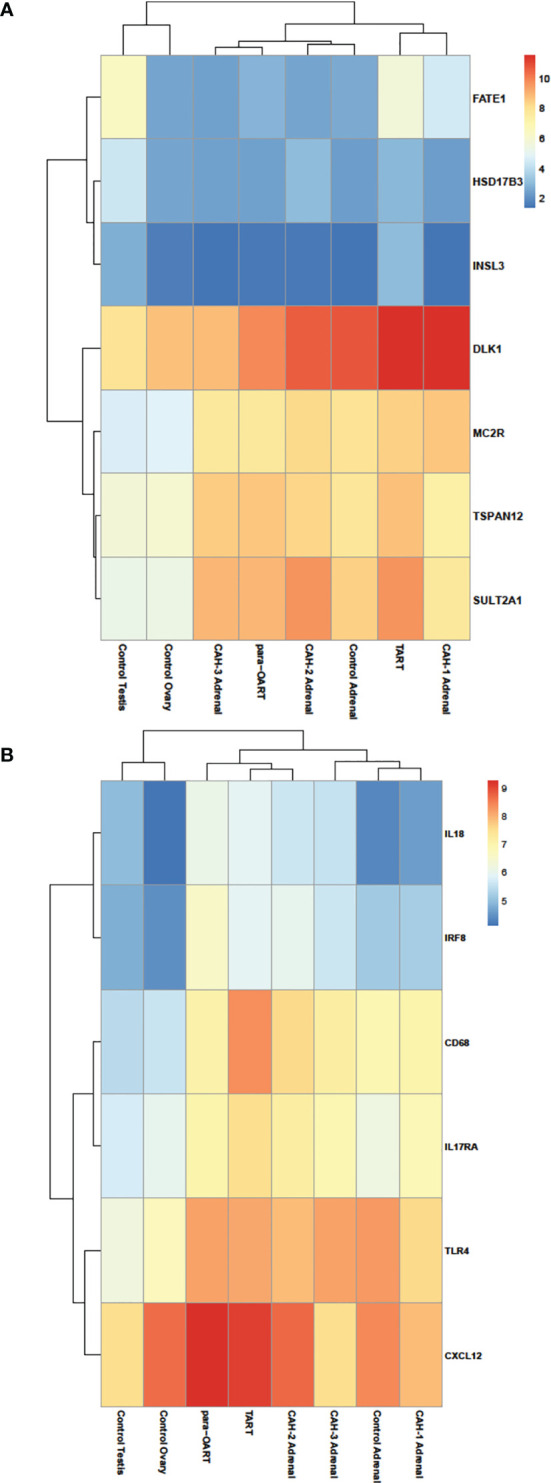
Heatmap illustrating differential RNA-seq expression data in ART, CAH adrenals and control tissues. Heatmap showing relative log-transformed expression levels of **(A)** adrenal enriched- (*DLK1, MC2R, TSPAN12, SULTA1*), gonadal enriched- (*FATE1, HSD17B3, INSL3*) markers, and **(B)** genes specific to inflammation and immune response (*IL18, IRF8, CD68, IL17RA, TLR4, CXCL12*). Red color indicates relatively higher number of transcripts and blue color indicates relatively lower number of transcripts based on normalized read counts.

Transcriptome differences between CAH adrenals and ART were compared to unaffected control tissue samples. RNA-seq differential expression data showed a high abundance of *MC2R, DLK1* transcripts in both the ART tissues (similar to control adrenals) and increased gonadal- specific *INSL3, FATE1, HSD17B3* transcripts in TART tissue (similar to control testis), along with an increase in ZR-specific *TSPAN12, SULTA1* transcripts in ART and CAH adrenals compared to control adrenals (**Figure 4A**). The transcriptional profile of CAH adrenals and ART showed a high abundance of inflammation related gene *IL18, IRF8, CD68, IL17RA, CXCL12* transcripts compared to control adrenals (**Figure 4B**).

**Figure 5:**
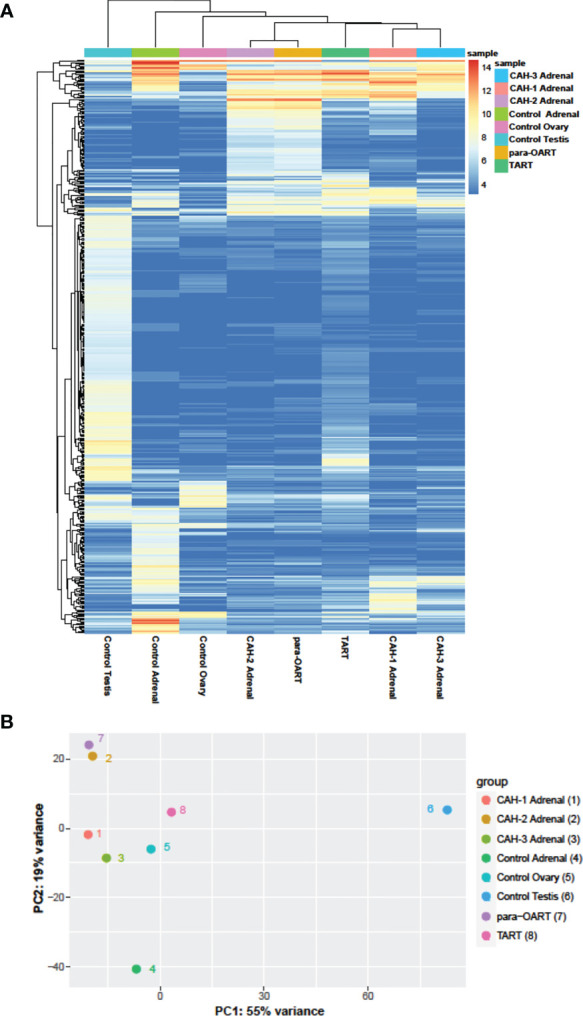
Hierarchical clustering and principle component analysis of ART, CAH adrenals and control tissues. **(A)** Heatmap of unsupervised clustering of the samples showing the top 500 most- variable genes, and **(B)** Principal Component Analysis (PCA) showing the correlation between the samples.

Unsupervised clustering revealed that para-OART was most similar to CAH adrenals compared to controls (adrenal and ovary). Similarly, TART was most similar to CAH adrenals and least similar to control testis (**Figures 5A, B**). Para-OART and adrenal tissue from the same CAH patient was clustered together and minimal deviation was observed between the other CAH adrenals. Control testis was clustered separately. Principle component analysis (PCA) confirmed that para-OART was very similar to the CAH adrenal of the same patient. The ART transcriptomes were most similar to the CAH adrenals, and least similar to the control testis followed by the control adrenal sample (**Figure 5B**). There is some variation between the two ART samples, where one, para-OART, showed more variance with the control adrenal and ovary and the other, TART, showing less variance with control ovary (**Figures 5A, B**). In summary, based on the unsupervised clustering methods and PCA, TART and para-OART were more similar to CAH adrenals than to the control adrenal and TART was less similar to the control testis than para-OART was to the control ovary.

## Discussion

Tumor formation is a common complication in CAH and TART is the most common cause of infertility in men with classic CAH (13, 14). Knowledge about the morphology of CAH adrenals and ART is important in developing treatment strategies and previous studies of TART have focused on adrenal-, testis- and fetal progenitor-like characteristics (35, 36). This is the first study to systematically characterize the adrenal glands of patients with CAH and ART, and compare to control adrenal and gonadal tissues. We found that morphologically and histologically ART is more similar to CAH adrenals than unaffected control adrenals and TART is least similar to control testis. We also show that ART and CAH adrenals have a similar predominance of ZR-like tissue infiltrated with lymphocytes.

As expected, the CAH-affected adrenal glands displayed disorganized hyperplasia with a lack of defined adrenocortical zonation and incomplete medulla formation, as previously described (37). Histologically, both ART and CAH adrenals were similar and adrenal-specific immunostaining and transcriptional expression of adrenal-specific genes were also similar indicating that the morphology and characteristics of the cells residing in both tissues are comparable. Interestingly, three out of the five CAH adrenals had complete lack of zonation, and all had predominance of ZR-like tissue composed of CYB5A-positive cells. In normal young adult adrenals, the ZF and ZR are clearly segregated. Our finding of CYB5A immunoreactivity throughout the CAH adrenals is interesting and novel and is different than the expected finding of CYB5A restriction to the ZR observed in the control. Similarly, Turcu et al. found that CAH adrenals exhibited areas containing a mixture of HSD3B2 and CYB5A immunoreactivities, which are typically restricted to the ZF and ZR respectively (38). Our finding of low prevalence of CYP11B1 in CAH tissues (CAH adrenals and ART), was surprising since CYP11B1 plays a key role in the production of 11-oxygenated 19-carbon androgens, major adrenal androgens in classic CAH (39). However, we saw clear evidence of the presence of CYP11B1 in CAH adrenals and ART. The overall percent of cells staining positive for CYP11B1 was low on any given section-likely because the patients had adrenal hyperplasia and increased ZR, accounting for the overall low CYP11B1 presence. The extent of adrenal disorganization was not associated with genotype/phenotype as two patients with classic simple-virilizing CAH with history of chronic non-compliance to hormone replacement therapy had a lack of adrenal zonation, suggesting that years of stimulus by elevated ACTH played an important role in altering tissue morphology.

Normally, in young adults, the adrenal cortex consists of an outer thin ZG covering approximately 15% of the cortex, a middle extensive ZF spanning approximately 75% and an innermost ZR covering approximately 10% of the cortical region (40–42). Our results from both histology and immunohistochemistry reveal dramatic hyperplasia of the ZR of CAH patients covering approximately 90 to 95% of the cortex, reflecting ZR cell proliferation and hyperplasia. Investigating the cell senescence pattern in adrenocortical zones, especially in ZR of CAH adrenals, might shed more light on this finding.

Prior studies show that expression of CYB5A, an adrenal ZR marker reflecting the presence of androgen production, increases with age during childhood corresponding to adrenarche, a time of physiological increase in adrenal androgen production and expansion of the ZR (43, 44). Moreover, in the ZR, both 17-hydroxylase and 17,20 lyase activity are present, resulting in efficient production of sex steroids. The rate of 17,20 lyase activity can be increased more than 10-fold by the presence of CYB5A (45) and mutations in *CYB5A* are associated with 17,20-lyase deficiency (46). Thus, our findings provide insight into the apparent efficient conversion of 17-hydroxyprogesterone to androstenedione observed in patients with CAH in poor control, while only minimal amounts of 17-hydroxyprogesterone are converted to androstenedione in the normal adrenal (47). As previously shown, CYB5A is expressed in many steroidogenic tissues (48–50) and plays a role in androgen production, therefore our findings of positive CYB5A staining in Leydig cells of control testis and control ovary was not surprising.

Although the etiology of ART and factors contributing to its onset and progression are not completely understood, there are different postulated theories. Because of the morphological, biochemical and histological similarities of TART with fetal adrenal cells, Lottrup et al. hypothesized that TART is derived from displaced adrenal cells which descended along with the testes during embryonic development (35). As TART has also been shown to exhibit testicular characteristics, others have suggested that TART may develop from pluripotent cells within the testes exhibiting both adrenal and Leydig cell features as shown by Smeets et al. (36). DLK1, a noncanonical notch receptor ligand is secreted by endocrine cells (51), and is a marker of immature Leydig cells (52). The number of cells expressing DLK1 is increased during fetal development, and it has also been proposed as a marker of regenerative potential (53). We found DLK1 expression in mature control Leydig and adrenal cells, in addition to CAH adrenal and ART, thus DLK1 did not help differentiate the origin of ART. The pathophysiology of TART development is elusive but ACTH receptors are present in TART (2) and poor hormonal control is known to promote tumor growth in patients with CAH (37, 54).

Furthermore, the presence of luteinizing hormone receptor (LHCGR) gene expression in TART and the increased prevalence of TART in adolescence, suggest that the pubertal rise in LH may also play a role in stimulating or promoting growth of the already present adrenal rest cells (15, 28, 36, 55, 56). Although the *in utero* environment might influence TART development, TART also rarely occurs with chronic ACTH elevation associated with acquired conditions (25, 29, 57) supporting the theory that TART originates from totipotent embryonic cells which grow when exposed to increased ACTH *in utero* (58) and early in life. However, TARTs from CAH patients and from patients with Cushing’s disease were recently shown to have similar characteristics despite the different timing of excessive ACTH exposure (59). Using transcriptional analysis, Schröder et al. showed higher transcriptional similarities of TART with adult adrenal tissues compared to fetal tissues suggesting that TART originates from a more distinct cell type rather than from a totipotent embryonic cell type (59). Our data from both IHC and transcriptomal profiling indicate that ART has both adrenal and gonadal-specific characteristics, supporting the theory that TART may originate from a totipotent cell with multi-differentiation ability. However, the clustering analysis and principal component analysis (PCA) data from our study provide evidence that ART tissues are most similar to CAH adrenals. These results suggests that ART may have originated from a totipotent cell or a more distinct cell type, but most importantly, suggest that both CAH-affected adrenal cells and ART respond similarly, as they are exposed to similar hormonal milieu including chronic ACTH excess.

Morphologically ART lesions were composed of eosinophilic cells resembling adrenocortical cells, with a benign, lymphoid infiltrate. In para-OART, nodular lymphocytic aggregates with lymphoid germinal centers were observed and lymphocytic aggregates in the adrenal parenchyma were observed in three out of five CAH adrenals. It is possible that years of glucocorticoid deficiency contributed to the development of these lymphocytic aggregates as all patients had years of noncompliance or undertreatment. The presence of these lymphocytes, especially the B-lymphocytes, suggests that they could play a role in tumor pathogenesis. Transcriptomal data also revealed an increase in expression of proinflammatory cytokines and cell surface markers on inflammatory cells. Studies exploring immune-endocrine interactions demonstrate that immune cells, especially lymphocytes, are present in the ZR and may play a role in the regulation of androgen production (60). With age, immune cells infiltrate the adrenal gland and influence the function of various zones by interacting with the adrenal cells (61). However, the possible role of inflammation in stimulating the growth of these tissues is unknown.

Importantly, our study emphasizes the high similarity between CAH adrenals and ART (both TART and para-OART) compared to control adrenals and gonads. The greatest advantage of our study was the inclusion of adrenals from CAH patients, which allowed us to focus on disease-specific effects. Our findings of similar patterns of a marked increase in adrenocortical and zone characteristic markers, along with the presence of nodular lymphocytic infiltration in ART and CAH adrenals, demonstrates that ART and CAH-affected adrenals originate from and differentiate into similar cell types. The main limitation of our study was the small sample size, especially the small number of adrenal rest and control tissues. The inclusion of one TART is a major limitation, especially since the patient had nonclassic or partial P450scc deficiency, a rare type of CAH. Despite the fact that this patient did not have 21-hydroxylase deficiency, he was similarly exposed to prolonged elevation of ACTH, providing insight into TART formation no matter what the cause of the ACTH elevation. We also have a biased population because only those patients in poor control with prolonged exposure to elevated ACTH and an abnormal hormonal environment underwent adrenal surgery. Though various techniques were used in the study, further studies should be performed with larger sample size.

In conclusion, our study represents a comparative histological and molecular characterization of both adrenal glands and ART affected by CAH. We found that ART is most similar to CAH-affected adrenal tissue compared to control adrenal and gonadal tissue, suggesting that both adrenal tissue and ART are similarly affected by the abnormal hormonal milieu. We also found predominance of ZR in hyperplastic CAH adrenals, possibly explaining the efficient adrenal androgen production observed in many patients with CAH. Characterization of CAH adrenals and ART is crucial to the development of future therapeutic interventions.

## Data Availability Statement 

The RNA-seq datasets generated for this study can be found in the National Center for Biotechnology Information BioProject, https://www.ncbi.nlm.nih.gov/bioproject/PRJNA761730 (BioProject number PRJNA761730).

## Ethics Statement

The studies involving human participants were reviewed and approved by National Institutes of Health (NIH) Institutional Review Board. Written informed consent to participate in this study was provided by the participants’ legal guardian/next of kin.

## Author Contributions

VK supervised, designed the experiments and analyzed the data, drafted, reviewed the manuscript. IW, SK, and MQ participated in design, interpretation of the IHC experiments, reviewed and edited the manuscript. JI, TL, and SC performed and participated in interpretation of RNA-seq data, reviewed and edited manuscript. AM and AG participated in data collection and interpretation of results and contributed to drafting the article. DM participated in data collection, supervised the project and manuscript writing. All authors contributed to the article and approved the submitted version.

## Funding

This study was funded by the intramural research program of the National Institutes of Health. Outside funding did not play a role in this study. Study design, collection, analysis, interpretation of data, and writing the manuscript was performed by the authors as federal employees of the National Institutes of Health.

## Conflict of Interest

DM received unrelated research funds from Diurnal Limited through National Institutes of Health Cooperative Research and Development Agreement.

The remaining authors declare that the research was conducted in the absence of any commercial or financial relationships that could be construed as a potential conflict of interest.

## Publisher’s Note

All claims expressed in this article are solely those of the authors and do not necessarily represent those of their affiliated organizations, or those of the publisher, the editors and the reviewers. Any product that may be evaluated in this article, or claim that may be made by its manufacturer, is not guaranteed or endorsed by the publisher.

## Supplementary Material

Supplementary Figure 1 Immunolabeling of DLK1, CYB5A in control adrenal, testis and ovary. Immunohistochemical staining showing high resolution of DLK1, CYB5A staining for control adrenal, testis and ovary. In the control adrenal (original magnification X40) DLK1 is positive, and strongest in the cells under the capsule and the ZG where cell proliferation is high. DLK1 is mildly stained in control testis, and negative in the control ovary (original magnification X200). Low magnification (original magnification X40) of the control adrenal shows the entire adrenal cortex with strong positive CYB5A staining of the zona reticularis.CYB5A staining is also strongly positive in Leydig cells (arrow) in the control testis and medium to strong positive in the control ovary (Original magnification X200).

## References

[1] Merke DP Auchus RJ. Congenital Adrenal Hyperplasia Due to 21-Hydroxylase Deficiency. N Engl J Med (2020) 383(13):1248–61. doi: 10.1056/NEJMra1909786 32966723

[2] Claahsen-van der Grinten HL Otten BJ Sweep FC Span PN Ross HA Meuleman EJ. Testicular Tumors in Patients With Congenital Adrenal Hyperplasia Due to 21-Hydroxylase Deficiency Show Functional Features of Adrenocortical Tissue. J Clin Endocrinol Metab (2007) 92(9):3674–80. doi: 10.1210/jc.2007-0337 17595257

[3] Claahsen-van der Grinten HL Hermus AR Otten BJ. Testicular Adrenal Rest Tumours in Congenital Adrenal Hyperplasia. Int J Pediatr Endocrinol (2009) 2009:624823. doi: 10.1186/1687-9856-2009-624823 19956703 PMC2777016

[4] Tajima T Funakoshi A Ikeda Y Hachitanda Y Yamaguchi M Yokota M. Nonfunctioning Adrenal Rest Tumor of the Liver: Radiologic Appearance. J Comput Assist Tomogr (2001) 25(1):98–101. doi: 10.1097/00004728-200101000-00018 11176302

[5] Skórka A Moszczyńska E Kot K Roszkowski M Jurkiewicz E Grajkowska W. Ectopic Virilising Adrenocortical Tumour in the Spinal Region in an 8 Year-Old Boy: A Case Report and Review of the Literature. Ital J Pediatr (2015) 41(1):4. doi: 10.1186/s13052-015-0169-8 26329697 PMC4557221

[6] Kepes JJ O'Boynick P Jones S Baum D McMillan J Adams ME. Adrenal Cortical Adenoma in the Spinal Canal of an 8-Year-Old Girl. Am J Surg Pathol (1990) 14(5):481–4. doi: 10.1097/00000478-199005000-00008 2327553

[7] Falls JL. Accessory Adrenal Cortex in the Broad Ligament: Incidence and Functional Significance. Cancer (1955) 8(1):143–50. doi: 10.1002/1097-0142(1955)8:1<143::aid-cncr2820080120>3.0.co;2-p 13231045

[8] Stikkelbroeck NM Hermus AR Schouten D Suliman HM Jager GJ Braat DD. Prevalence of Ovarian Adrenal Rest Tumours and Polycystic Ovaries in Females With Congenital Adrenal Hyperplasia: Results of Ultrasonography and MR Imaging. Eur Radiol (2004) 14(10):1802–6. doi: 10.1007/s00330-004-2329-x 15322809

[9] Sisto JM Liu FW Geffner ME Berman ML. Para-Ovarian Adrenal Rest Tumors: Gynecologic Manifestations of Untreated Congenital Adrenal Hyperplasia. Gynecol Endocrinol (2018) 34(8):644–6. doi: 10.1080/09513590.2018.1441399 29460643

[10] Thomas TT Ruscher KR Mandavilli S Balarezo F Finck CM. Ovarian Steroid Cell Tumor, Not Otherwise Specified, Associated With Congenital Adrenal Hyperplasia: Rare Tumors of an Endocrine Disease. J Pediatr Surg (2013) 48(6):E23–7. doi: 10.1016/j.jpedsurg.2013.04.006 23845653

[11] Claahsen-van der Grinten HL Hulsbergen-van de Kaa CA Otten BJ. Ovarian Adrenal Rest Tissue in Congenital Adrenal Hyperplasia–A Patient Report. J Pediatr Endocrinol Metab (2006) 19(2):177–82. doi: 10.1515/JPEM.2006.19.2.177 16562593

[12] Crocker MK Barak S Millo CM Beall SA Niyyati M Chang R. Use of PET/CT With Cosyntropin Stimulation to Identify and Localize Adrenal Rest Tissue Following Adrenalectomy in a Woman With Congenital Adrenal Hyperplasia. J Clin Endocrinol Metab (2012) 97(11):E2084–9. doi: 10.1210/jc.2012-2298 PMC3485588 22904181

[13] Claahsen-van der Grinten HL Otten BJ Hermus AR Sweep FC Hulsbergen-van de Kaa CA. Testicular Adrenal Rest Tumors in Patients With Congenital Adrenal Hyperplasia Can Cause Severe Testicular Damage. Fertil Steril (2008) 89(3):597–601. doi: 10.1016/j.fertnstert.2007.03.051 17543962

[14] Stikkelbroeck NM Otten BJ Pasic A Jager GJ Sweep CG Noordam K. High Prevalence of Testicular Adrenal Rest Tumors, Impaired Spermatogenesis, and Leydig Cell Failure in Adolescent and Adult Males With Congenital Adrenal Hyperplasia. J Clin Endocrinol Metab (2001) 86(12):5721–8. doi: 10.1210/jcem.86.12.8090 11739428

[15] Claahsen-van der Grinten HL Dehzad F Kamphuis-van Ulzen K de Korte CL. Increased Prevalence of Testicular Adrenal Rest Tumours During Adolescence in Congenital Adrenal Hyperplasia. Horm Res Paediatr (2014) 82(4):238–44. doi: 10.1159/000365570 25195868

[16] Engels M Span PN van Herwaarden AE Sweep F Stikkelbroeck N Claahsen-van der Grinten HL. Testicular Adrenal Rest Tumors: Current Insights on Prevalence, Characteristics, Origin, and Treatment. Endocr Rev (2019) 40(4):973–87. doi: 10.1210/er.2018-00258 30882882

[17] Mazzilli R Stigliano A Delfino M Olana S Zamponi V Iorio C. The High Prevalence of Testicular Adrenal Rest Tumors in Adult Men With Congenital Adrenal Hyperplasia Is Correlated With ACTH Levels. Front Endocrinol (Lausanne) (2019) 10:335. doi: 10.3389/fendo.2019.00335 31214118 PMC6558150

[18] Rutgers JL Young RH Scully RE. The Testicular "Tumor" of the Adrenogenital Syndrome. A Report of Six Cases and Review of the Literature on Testicular Masses in Patients With Adrenocortical Disorders. Am J Surg Pathol (1988) 12(7):503–13. doi: 10.1097/00000478-198807000-00001 3291624

[19] Wang Z Yang S Shi H Du H Xue L Wang L. Histopathological and Immunophenotypic Features of Testicular Tumour of the Adrenogenital Syndrome. Histopathology (2011) 58(7):1013–8. doi: 10.1111/j.1365-2559.2011.03861.x 21707702

[20] Clark RV Albertson BD Munabi A Cassorla F Aguilera G Warren DW. Steroidogenic Enzyme Activities, Morphology, and Receptor Studies of a Testicular Adrenal Rest in a Patient With Congenital Adrenal Hyperplasia. J Clin Endocrinol Metab (1990) 70(5):1408–13. doi: 10.1210/jcem-70-5-1408 2335578

[21] O'Shaughnessy PJ Baker PJ Johnston H. The Foetal Leydig Cell– Differentiation, Function and Regulation. Int J Androl (2006) 29(1):90–5; discussion 105–8. doi: 10.1111/j.1365-2605.2005.00555.x 16466528

[22] Val P Jeays-Ward K Swain A. Identification of a Novel Population of Adrenal-Like Cells in the Mammalian Testis. Dev Biol (2006) 299(1):250–6. doi: 10.1016/j.ydbio.2006.07.030 16949566

[23] Hamwi GJ Gwinup G Mostow JH Besch PK. Activation of Testicular Adrenal Rest Tissue by Prolonged Excessive Acth Production. J Clin Endocrinol Metab (1963) 23:861–9. doi: 10.1210/jcem-23-9-861 14064131

[24] Claahsen-van der Grinten HL Otten BJ Sweep FC Hermus AR. Repeated Successful Induction of Fertility After Replacing Hydrocortisone With Dexamethasone in a Patient With Congenital Adrenal Hyperplasia and Testicular Adrenal Rest Tumors. Fertil Steril (2007) 88(3):705.e5–8. doi: 10.1016/j.fertnstert.2006.11.148 17517401

[25] Puar T Engels M van Herwaarden AE Sweep FC Hulsbergen-van de Kaa C Kamphuis-van Ulzen K. Bilateral Testicular Tumors Resulting in Recurrent Cushing Disease After Bilateral Adrenalectomy. J Clin Endocrinol Metab (2017) 102(2):339–44. doi: 10.1210/jc.2016-2702 27901643

[26] Yu MK Jung MK Kim KE Kwon AR Chae HW Kim DH. Clinical Manifestations of Testicular Adrenal Rest Tumor in Males With Congenital Adrenal Hyperplasia. Ann Pediatr Endocrinol Metab (2015) 20(3):155–61. doi: 10.6065/apem.2015.20.3.155 PMC4623344 26512352

[27] Finkielstain GP Kim MS Sinaii N Nishitani M Van Ryzin C Hill SC. Clinical Characteristics of a Cohort of 244 Patients With Congenital Adrenal Hyperplasia. J Clin Endocrinol Metab (2012) 97(12):4429–38. doi: 10.1210/jc.2012-2102 PMC3513542 22990093

[28] Mouritsen A Jorgensen N Main KM Schwartz M Juul A. Testicular Adrenal Rest Tumours in Boys, Adolescents and Adult Men With Congenital Adrenal Hyperplasia May Be Associated With the CYP21A2 Mutation. Int J Androl (2010) 33(3):521–7. doi: 10.1111/j.1365-2605.2009.00967.x 19531083

[29] Ashley RA McGee SM Isotaolo PA Kramer SA Cheville JC. Clinical and Pathological Features Associated With the Testicular Tumor of the Adrenogenital Syndrome. J Urol (2007) 177(2):546–9; discussion 9. doi: 10.1016/j.juro.2006.09.041 17222630

[30] Ntalles K Kostoglou-Athanassiou I Georgiou E Ikkos D. Paratesticular Tumours in a Patient With Nelson's Syndrome. Horm Res (1996) 45(6):291–4. doi: 10.1159/000184808 8793524

[31] El-Maouche D Hargreaves CJ Sinaii N Mallappa A Veeraraghavan P Merke DP. Longitudinal Assessment of Illnesses, Stress Dosing, and Illness Sequelae in Patients With Congenital Adrenal Hyperplasia. J Clin Endocrinol Metab (2018) 103(6):2336–45. doi: 10.1210/jc.2018-00208 PMC6276663 29584889

[32] Mallappa A Millo CM Quezado M Merke DP. Congenital Adrenal Hyperplasia Presenting as an Adrenal Mass With Increased (18)F-FDG Positron Emission Tomography Uptake. J Endocr Soc (2017) 1(8):1110–2. doi: 10.1210/js.2017-00270 PMC5686602 29264564

[33] Dagalakis U Mallappa A Elman M Quezado M Merke DP. Positive Fertility Outcomes in a Female With Classic Congenital Adrenal Hyperplasia Following Bilateral Adrenalectomy. Int J Pediatr Endocrinol (2016) 2016:10. doi: 10.1186/s13633-016-0028-4 27212956 PMC4873998

[34] Kolli V Kim H Torky A Lao Q Tatsi C Mallappa A. Characterization of the CYP11A1 Nonsynonymous Variant P.E314K in Children Presenting With Adrenal Insufficiency. J Clin Endocrinol Metab (2019) 104(2):269–76. doi: 10.1210/jc.2018-01661 PMC6607962 30299480

[35] Lottrup G Nielsen JE Skakkebaek NE Juul A Rajpert-De Meyts E. Abundance of DLK1, Differential Expression of CYP11B1, CYP21A2 and MC2R, and Lack of INSL3 Distinguish Testicular Adrenal Rest Tumours From Leydig Cell Tumours. Eur J Endocrinol (2015) 172(4):491–9. doi: 10.1530/EJE-14-0810 25609776

[36] Smeets EE Span PN van Herwaarden AE Wevers RA Hermus AR Sweep FC. Molecular Characterization of Testicular Adrenal Rest Tumors in Congenital Adrenal Hyperplasia: Lesions With Both Adrenocortical and Leydig Cell Features. J Clin Endocrinol Metab (2015) 100(3):E524–30. doi: 10.1210/jc.2014-2036 25485724

[37] Merke DP Chrousos GP Eisenhofer G Weise M Keil MF Rogol AD. Adrenomedullary Dysplasia and Hypofunction in Patients With Classic 21-Hydroxylase Deficiency. N Engl J Med (2000) 343(19):1362–8. doi: 10.1056/NEJM200011093431903 11070100

[38] Turcu AF Nanba AT Chomic R Upadhyay SK Giordano TJ Shields JJ. Adrenal-Derived 11-Oxygenated 19-Carbon Steroids Are the Dominant Androgens in Classic 21-Hydroxylase Deficiency. Eur J Endocrinol (2016) 174(5):601–9. doi: 10.1530/EJE-15-1181 PMC4874183 26865584

[39] Turcu AF Auchus RJ. Clinical Significance of 11-Oxygenated Androgens. Curr Opin Endocrinol Diabetes Obes (2017) 24(3):252–9. doi: 10.1097/MED.0000000000000334 PMC5819755 28234803

[40] Hui XG Akahira J Suzuki T Nio M Nakamura Y Suzuki H. Development of the Human Adrenal Zona Reticularis: Morphometric and Immunohistochemical Studies From Birth to Adolescence. J Endocrinol (2009) 203(2):241–52. doi: 10.1677/JOE-09-0127 PMC4159054 19723922

[41] Yates R Katugampola H Cavlan D Cogger K Meimaridou E Hughes C. Adrenocortical Development, Maintenance, and Disease. Curr Top Dev Biol (2013) 106:239–312. doi: 10.1016/B978-0-12-416021-7.00007-9 24290352

[42] Pignatti E Leng S Carlone DL Breault DT. Regulation of Zonation and Homeostasis in the Adrenal Cortex. Mol Cell Endocrinol (2017) 441:146–55. doi: 10.1016/j.mce.2016.09.003 PMC5235909 27619404

[43] Rege J Nakamura Y Wang T Merchen TD Sasano H Rainey WE. Transcriptome Profiling Reveals Differentially Expressed Transcripts Between the Human Adrenal Zona Fasciculata and Zona Reticularis. J Clin Endocrinol Metab (2014) 99(3):E518–27. doi: 10.1210/jc.2013-3198 PMC3942232 24423296

[44] Rege J Karashima S Lerario AM Smith JM Auchus RJ Kasa-Vubu JZ. Age-Dependent Increases in Adrenal Cytochrome B5 and Serum 5-Androstenediol-3-Sulfate. J Clin Endocrinol Metab (2016) 101(12):4585–93. doi: 10.1210/jc.2016-2864 PMC5155691 27623070

[45] Miller WL Auchus RJ. The Molecular Biology, Biochemistry, and Physiology of Human Steroidogenesis and Its Disorders. Endocr Rev (2011) 32(1):81–151. doi: 10.1210/er.2010-0013 21051590 PMC3365799

[46] Turcu AF Auchus RJ. The Next 150 Years of Congenital Adrenal Hyperplasia. J Steroid Biochem Mol Biol (2015) 153:63–71. doi: 10.1016/j.jsbmb.2015.05.013 26047556 PMC4568140

[47] Speiser PW Arlt W Auchus RJ Baskin LS Conway GS Merke DP. Congenital Adrenal Hyperplasia Due to Steroid 21-Hydroxylase Deficiency: An Endocrine Society Clinical Practice Guideline. J Clin Endocrinol Metab (2018) 103(11):4043–88. doi: 10.1210/jc.2018-01865 PMC6456929 30272171

[48] Dharia S Slane A Jian M Conner M Conley AJ Parker CR Jr. Colocalization of P450c17 and Cytochrome B5 in Androgen-Synthesizing Tissues of the Human. Biol Reprod (2004) 71(1):83–8. doi: 10.1095/biolreprod.103.026732 14985252

[49] Simard J Ricketts ML Gingras S Soucy P Feltus FA Melner MH. Molecular Biology of the 3beta-Hydroxysteroid Dehydrogenase/Delta5-Delta4 Isomerase Gene Family. Endocr Rev (2005) 26(4):525–82. doi: 10.1210/er.2002-0050 15632317

[50] Nakamura Y Xing Y Hui XG Kurotaki Y Ono K Cohen T. Human Adrenal Cells That Express Both 3beta-Hydroxysteroid Dehydrogenase Type 2 (HSD3B2) and Cytochrome B5 (CYB5A) Contribute to Adrenal Androstenedione Production. J Steroid Biochem Mol Biol (2011) 123(3-5):122–6. doi: 10.1016/j.jsbmb.2010.12.001 PMC4269365 21185375

[51] Jensen CH Krogh TN Hojrup P Clausen PP Skjodt K Larsson LI. Protein Structure of Fetal Antigen 1 (FA1). A Novel Circulating Human Epidermal-Growth-Factor-Like Protein Expressed in Neuroendocrine Tumors and Its Relation to the Gene Products of Dlk and Pg2. Eur J Biochem (1994) 225(1):83–92. doi: 10.1111/j.1432-1033.1994.00083.x 7925474

[52] Lottrup G Nielsen JE Maroun LL Moller LM Yassin M Leffers H. Expression Patterns of DLK1 and INSL3 Identify Stages of Leydig Cell Differentiation During Normal Development and in Testicular Pathologies, Including Testicular Cancer and Klinefelter Syndrome. Hum Reprod (2014) 29(8):1637–50. doi: 10.1093/humrep/deu124 24908673

[53] Floridon C Jensen CH Thorsen P Nielsen O Sunde L Westergaard JG. Does Fetal Antigen 1 (FA1) Identify Cells With Regenerative, Endocrine and Neuroendocrine Potentials? A Study of FA1 in Embryonic, Fetal, and Placental Tissue and in Maternal Circulation. Differentiation (2000) 66(1):49–59. doi: 10.1046/j.1432-0436.2000.066001049.x 10997592

[54] Giacaglia LR Mendonca BB Madureira G Melo KF Suslik CA Arnhold IJ. Adrenal Nodules in Patients With Congenital Adrenal Hyperplasia Due to 21-Hydroxylase Deficiency: Regression After Adequate Hormonal Control. J Pediatr Endocrinol Metab (2001) 14(4):415–9. doi: 10.1515/JPEM.2001.14.4.415 11327375

[55] Mendes-Dos-Santos CT Martins DL Guerra-Junior G Baptista MTM de-Mello MP de Oliveira LC. Prevalence of Testicular Adrenal Rest Tumor and Factors Associated With Its Development in Congenital Adrenal Hyperplasia. Horm Res Paediatr (2018) 90(3):161–8. doi: 10.1159/000492082 30149373

[56] Kim MS Goodarzian F Keenan MF Geffner ME Koppin CM De Filippo RE. Testicular Adrenal Rest Tumors in Boys and Young Adults With Congenital Adrenal Hyperplasia. J Urol (2017) 197(3 Pt 2):931–6. doi: 10.1016/j.juro.2016.09.072 PMC5449264 27840017

[57] Johnson RE Scheithauer B. Massive Hyperplasia of Testicular Adrenal Rests in a Patient With Nelson's Syndrome. Am J Clin Pathol (1982) 77(4):501–7. doi: 10.1093/ajcp/77.4.501 7072659

[58] Engels M Span PN Mitchell RT Heuvel J Marijnissen-van Zanten MA van Herwaarden AE. GATA Transcription Factors in Testicular Adrenal Rest Tumours. Endocr Connect (2017) 6(8):866–75. doi: 10.1530/EC-17-0215 PMC5682415 29038332

[59] Schröder MAM Sweep FCGJ van Herwaarden AE Rowan AE Korbie D Mitchell RT. Transcriptional Comparison of Testicular Adrenal Rest Tumors With Fetal and Adult Tissues. bioRxiv (2020) 2020.05.07.082313:3, 5–6. doi: 10.1101/2020.05.07.082313 PMC7613903 36047744

[60] Wolkersdorfer GW Lohmann T Marx C Schroder S Pfeiffer R Stahl HD. Lymphocytes Stimulate Dehydroepiandrosterone Production Through Direct Cellular Contact With Adrenal Zona Reticularis Cells: A Novel Mechanism of Immune-Endocrine Interaction. J Clin Endocrinol Metab (1999) 84(11):4220–7. doi: 10.1210/jc.84.11.4220 10566676

[61] Marx C Bornstein SR Wolkersdorfer GW. Cellular Immune-Endocrine Interaction in Adrenocortical Tissues. Eur J Clin Invest (2000) 30 (Suppl 3):1–5. doi: 10.1046/j.1365-2362.2000.0300s3001.x 11281360

