# Supplementary figures and images for: Morphologic and Molecular Characterization of Adrenals and Adrenal Rest Affected by Congenital Adrenal Hyperplasia
Source: Front Endocrinol (Lausanne). 2021 Sep 20;12:730947. doi: 10.3389/fendo.2021.730947 (PMC8488225; doi:10.3389/fendo.2021.730947)

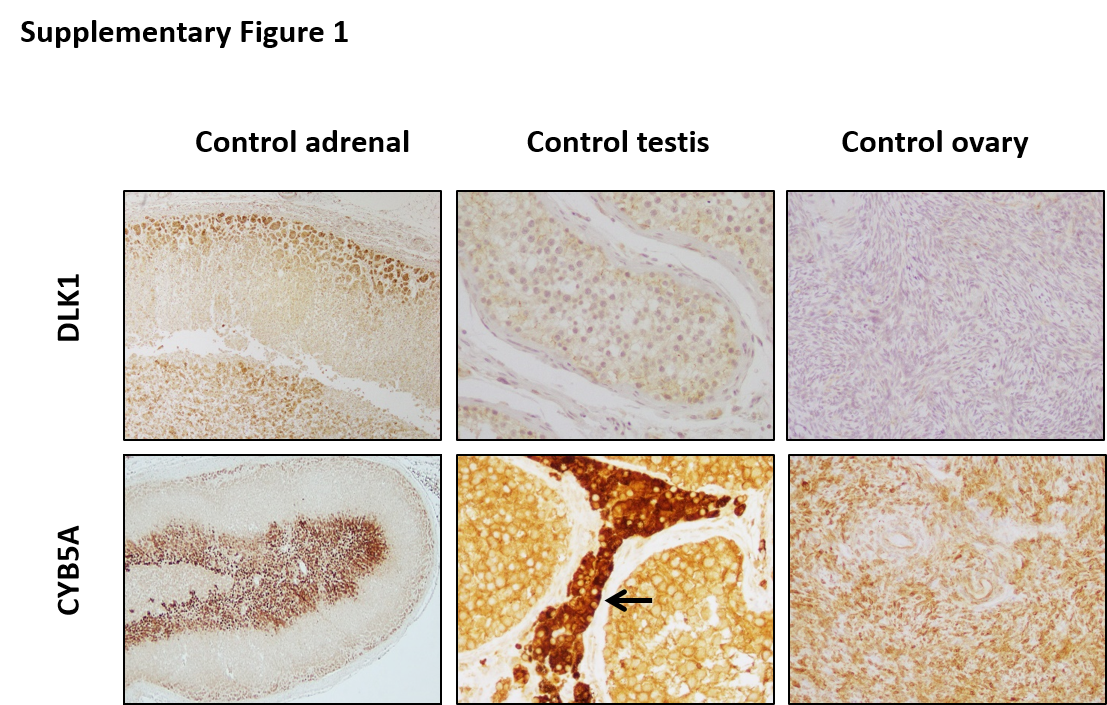

Supplement: Supplementary Figure 1 — Immunolabeling of DLK1, CYB5A in control adrenal, testis and ovary. Immunohistochemical staining showing high resolution of DLK1, CYB5A staining for control adrenal, testis and ovary. In the control adrenal (original magnification X40) DLK1 is positive, and strongest in the cells under the capsule and the ZG where cell proliferation is high. DLK1 is mildly stained in control testis, and negative in the control ovary (original magnification X200). Low magnification (original magnification X40) of the control adrenal shows the entire adrenal cortex with strong positive CYB5A staining of the zona reticularis.CYB5A staining is also strongly positive in Leydig cells (arrow) in the control testis and medium to strong positive in the control ovary (Original magnification X200). [file Image_1.tif]

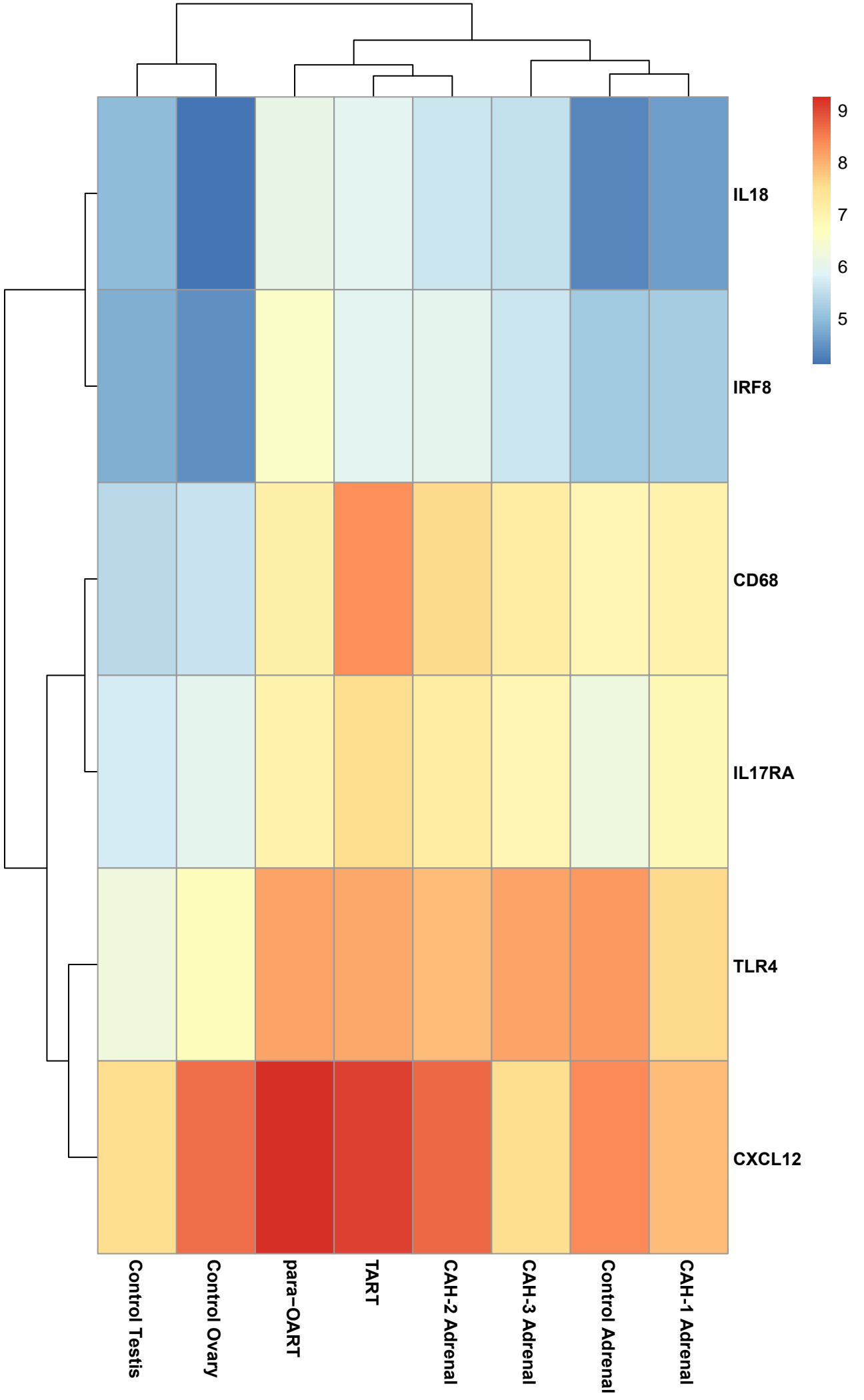

Supplement: Supplementary file 2 [file Image_2.pdf]

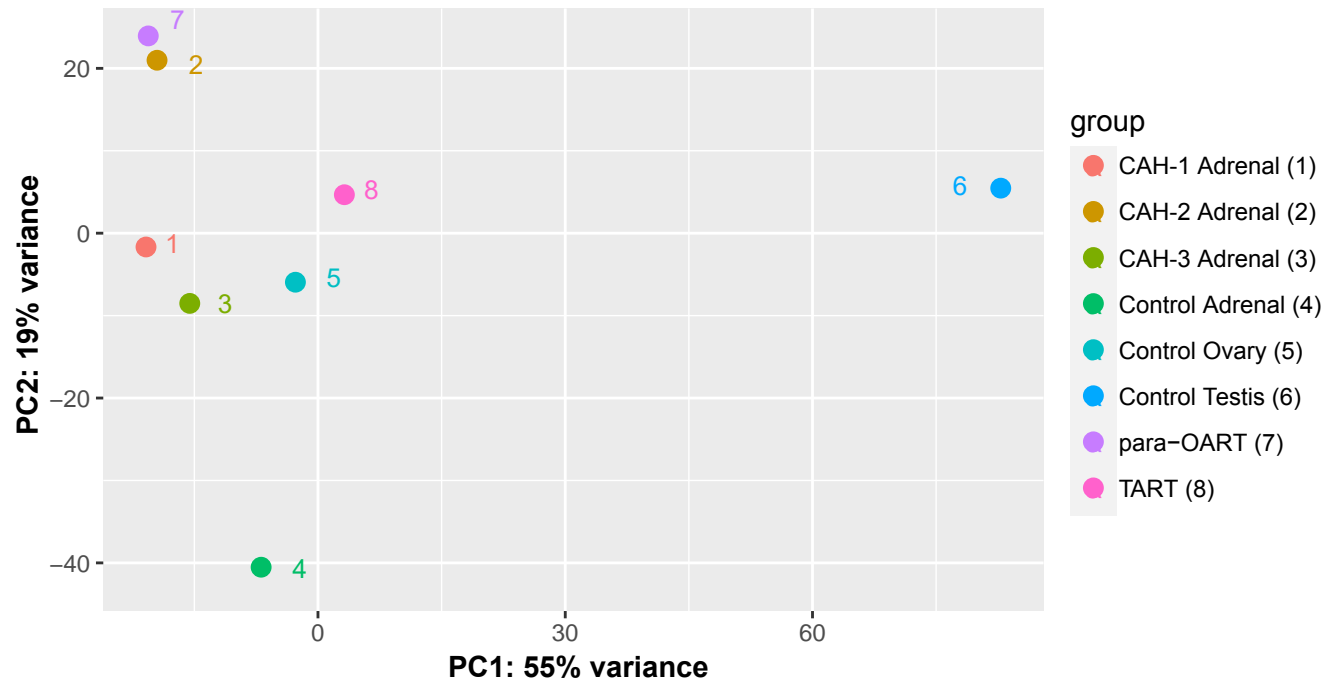

Supplement: Supplementary file 3 [file Image_3.pdf]

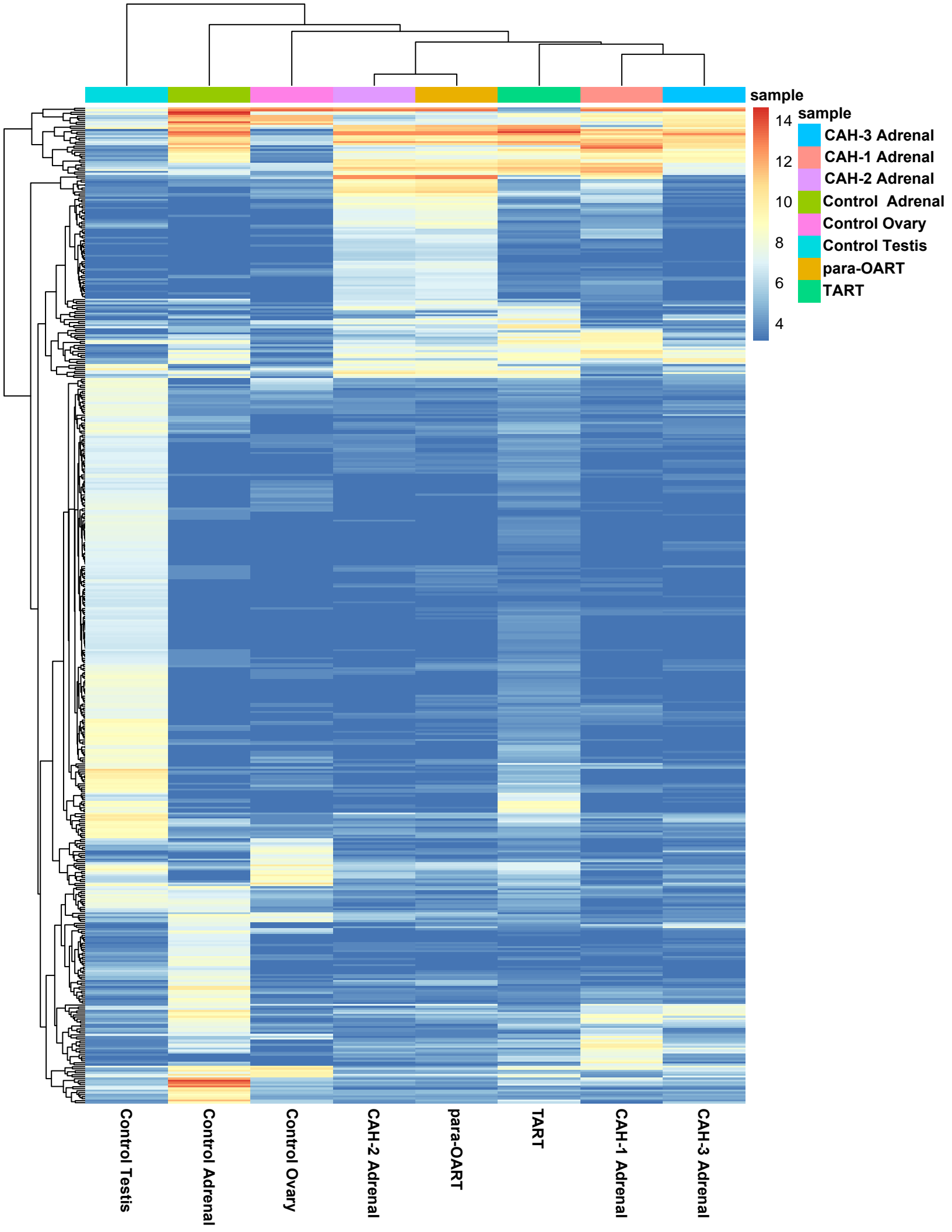

Supplement: Supplementary file 4 [file Image_4.pdf]

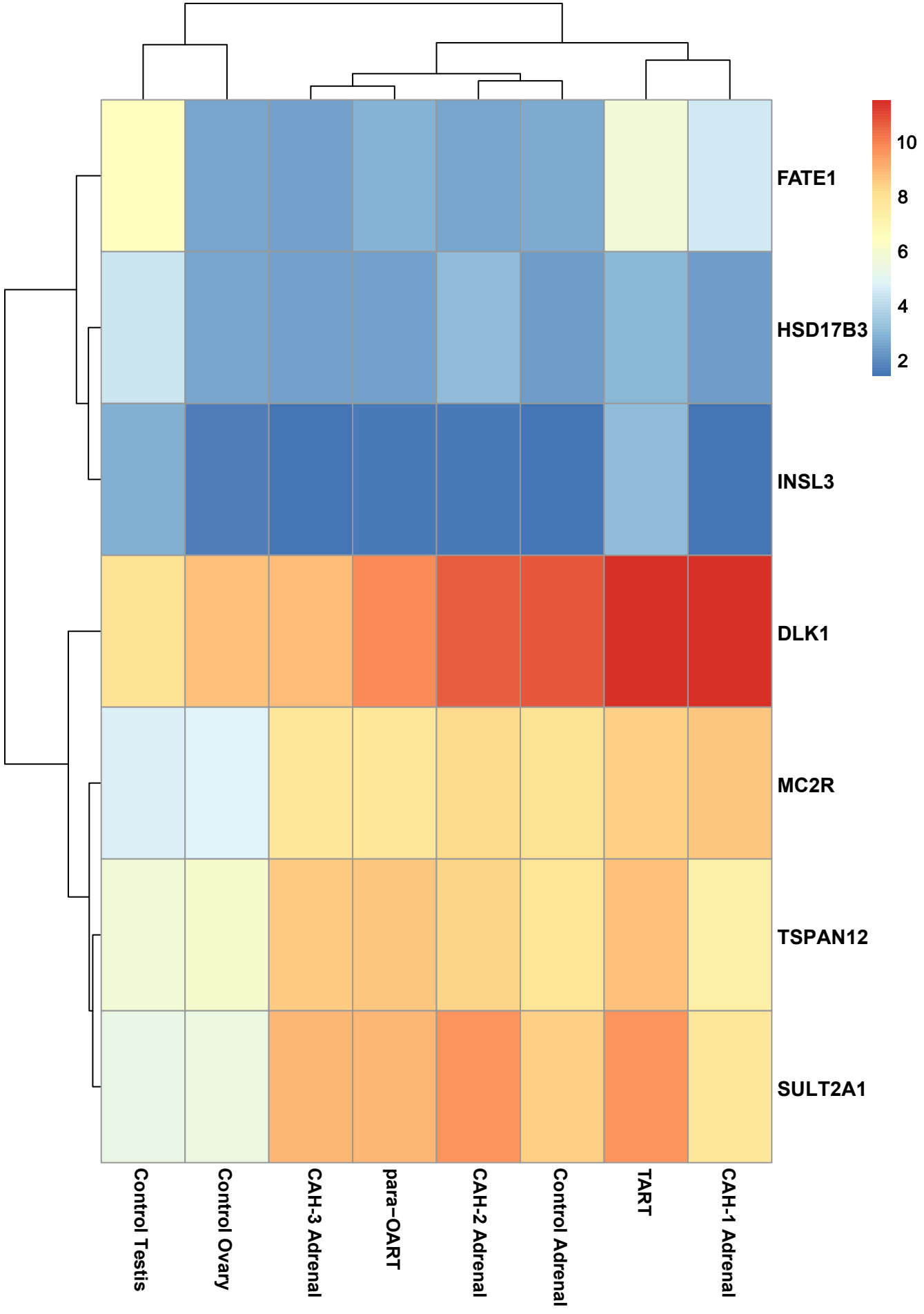

Supplement: Supplementary file 5 [file Image_5.pdf]
